# *Schizosaccharomyces pombe* Homologs of Human DJ-1 Are Stationary Phase-Associated Proteins That Are Involved in Autophagy and Oxidative Stress Resistance

**DOI:** 10.1371/journal.pone.0143888

**Published:** 2015-12-01

**Authors:** Yang Su, Caiping Chen, Linting Huang, Jianhua Yan, Ying Huang

**Affiliations:** Jiangsu Key Laboratory for Microbes and Functional Genetics, College of Life Science, Nanjing Normal University, Nanjing, China; University of Cambridge, UNITED KINGDOM

## Abstract

The Parkinson′s disease protein DJ-1 is involved in various cellular functions including detoxification of dicarbonyl compounds, autophagy and oxidative stress response. DJ-1 homologs are widely found in both prokaryotes and eukaryotes, constituting a superfamily of proteins that appear to be involved in stress response. *Schizosaccharomyces pombe* contains six DJ-1 homologs, designated Hsp3101-Hsp3105 and Sdj1 (previously named SpDJ-1). Here we show that deletion of any one of these six genes somehow affects autophagy during prolonged stationary phase. Furthermore, deletions of each of these DJ-1 homologs result in reduced stationary phase survival. Deletion of *sdj1* also increases the sensitivity of stationary-phase cells to oxidative stress induced by hydrogen peroxide (H_2_O_2_) whereas overexpression of *sdj1* has the opposite effect. Consistent with their role in stationary phase, expression of *hsp3101*, *hsp3102*, *hsp3105* and *sdj1*, and to a lesser extent *hsp3103* and *hsp3104*, is increased in stationary phase. The induction of *hsp3101*, *hsp3102*, *hsp3105* and *sdj1* involves the Sty1-regulated transcription factor Atf1 but not the transcription factor Pap1. Our results firmly establish that *S*. *pombe* homologs of DJ-1 are stationary-phase associated proteins and are likely involved in autophagy and antioxidant defense in stationary phase of *S*. *pombe* cells.

## Introduction

DJ-1 and Hsp31 proteins belong to two different subfamilies of the DJ-1/Hsp31/PfpI superfamily, which is composed of a wide variety of structurally similar but functionally diverse proteins [1–3]. Nearly all members of the DJ-1/Hsp31/PfpI superfamily have a conserved cysteine residue.

Human DJ-1 is a multifunctional protein implicated in cancer- and Parkinson’s disease (PD) [4–6]. Human DJ-1 has been found to modulate autophagy through the activation of the stress-activated JNK signaling pathway [7, 8]. Autophagy is a highly conserved catabolic process that degrades and recycles intracellular proteins and organelles to maintain energy homeostasis under nutrient-limiting conditions and to eliminate unwanted cellular material [9]. Besides, human DJ-1 is also a glutathione-independent glyoxalase (glyoxalase III) that detoxifies reactive dicarbonyl compounds such as glyoxal and methylglyoxal, which are toxic metabolites in living organisms [10]. It is generally believed that DJ-1 is primarily involved in resistance to oxidative stress and in protection against mitochondrial damage [11, 12]. However, the detailed molecular mechanisms remain to be elucidated.

The budding yeast *Saccharomyces cerevisiae* has four DJ-1 homologs, Hsp31, Hsp32, Hsp33, and Hsp34 (collectively called *S*. *cerevisiae* Hsp31 proteins), the latter three of which have nearly identical amino acid sequences. *S*. *cerevisiae* Hsp31 proteins exhibit extremely weak sequence similarity to DJ-1, indicating extraordinary degree of divergence between Hsp31 proteins and DJ-1. In contrast, these proteins all have significant sequence similarity to the *Escherichia coli* chaperone Hsp31 [13]. However, unlike *E*. *coli* Hsp31 which is not involved in oxidative stress response [14] and like human DJ-1, *S*. *cerevisiae* Hsp31 has a role in the protection against oxidative stress [15].

It has recently been shown that all *S*. *cerevisiae* Hsp31 proteins associate with autophagy [16]. Deletions of each individual of *S*. *cerevisiae HSP31* genes impair cell survival under nutrient-depleted environments and autophagy under carbon starvation. *S*. *cerevisiae* Hsp31 proteins appear to function upstream of the target of rapamycin complex 1 (TORC1) since deletion of *HSP31* disrupts localization of TORC1 to processing bodies (P-bodies), and causes abnormal TORC1-mediated phosphorylation of Atg13. Under nutrient-rich condition, TORC1 phosphorylation of Atg13 prevents its binding to the autophagy-related (Atg) protein Atg1. The binding of Atg1 to Atg13 is required for the kinase activity of Atg1, which is required for the induction of autophagy. In addition, *S*. *cerevisiae* Hsp31 functions as a glyoxalase III [17–20], a stress-responsive chaperone [21] and a protein deglycase [22].

We have identified six *Schizosaccharomyces pombe* DJ-1 homologs, designated Hsp3101-Hsp3105 (collectively called *S*. *pombe* Hsp31 proteins) and Sdj1 (previously named SpDJ-1) [19]. Unlike *S*. *cerevisiae* DJ-1 homologs, the sequences of *S*. *pombe* DJ-1 homologs are highly divergent; the percent identity varies from 10% to 58%. Among these genes, only Sdj1 exhibits a significant degree of sequence homology with human DJ-1 [19], whereas Hsp3101-Hsp3105 have significant sequence similarity to *E*. *coli* and *S*. *cerevisiae* Hsp31 proteins. We have recently shown that Hsp3101, Hsp3102 and Sdj1 are glyoxalase III enzymes and that the first two enzymes displayed significantly higher *in vitro* glyoxalase III activity than Sdj1 [19]. However, it is unclear whether *S*. *pombe* Hsp31 proteins and Sdj1 have a role in autophagy and oxidative stress.

In this study, we show that *S*. *pombe* DJ-1 homologs are somehow involved in autophagy and that Sdj1 is involved in oxidative stress. We also demonstrate that expression of *S*. *pombe* DJ-1 homologs are induced in stationary phase and that the induction is dependent on the mitogen-activated protein (MAP) kinase Sty1.

## Materials and Methods

### Strains and media

*S*. *pombe* strains used in this study are listed in Table 1. Strain DY3510 expressing N-terminal CFP tagged Atg8 (CFP-Atg8) under the control of its endogenous promoter was obtained from L. L. Du. Strain ySY1 expressing N-terminal GFP tagged Atg8 (GFP-Atg8) under the control of its endogenous promoter was constructed as described [23]. The null mutants for DJ-1 homologs in *S*. *pombe* were constructed by one-step gene replacement [24]. For phenotypic analysis, each DJ-1 homolog was deleted from the wild-type *S*. *pombe* strain yHL6381 as described [19]. To assess the role of *S*. *pombe* DJ-1 homologs in autophagy, each DJ-1 homolog was deleted from DY3510 (for the CFP-Atg8 processing assay) or ySY1 (for fluorescence microscopy analysis). Briefly, the deletion cassettes for *hsp3101* and *hsp3103* were constructed by cloning the 5′ and 3′ flanks of the genes into the *Spe*I-*Pst*I and the *Sal*I-*Kpn*I sites pAF1-KaMX6 (in which the *his3* ^+^ selectable marker in pAF1 [25] was replaced by the KanMX6 marker), respectively. The deletion cassettes for *hsp3102* and *sdj1* were constructed by cloning the 5′ and 3′ flanks of the genes into *Sma*I-*Bgl*II and *Pme*I-*EcoR*I sites of pFA6a-kanMX6 [26], respectively. The deletion cassette for *hsp3103* was constructed by cloning the 5′ and 3′ flanks of the gene into the *Spe*I-*Pst*I and *Sac*I-*EcoR*I sites of pFA6a-kanMX6, respectively. The deletion cassette for *hsp3104* were constructed by cloning the 5′ and 3′ flanks of the gene into the *Sal*I-*Bgl*II and *Sac*I-*EcoR*I sites of pFA6a-kanMX6, respectively. To generate Δ*atg5* deletion mutants which serve as negative controls in assays of autophagy, the *atg5* deletion cassette containing the KanMX6 selection marker flanked by the 5′ and 3′ flanks of *atg5* was obtained by PCR using genomic DNA isolated from the Δ*atg5* deletion mutant Zd299 (obtained from obtained from L. L. Du) as a template. The *atg5* deletion cassette was transformed into either DY3510 to generate ySY14, which is a negative control in the CFP-Atg8 processing assay, or ySY1 to generate ySY15, which is a negative control for fluorescence microscopy analysis. To generate Δ*sty1* and Δ*atf1* deletion mutants, the *sty1* and *atf1* deletion cassettes containing the *ura4* selection marker flanked by the 5′ and 3′ flanks of target genes were constructed by overlapping PCR. To generate strains expressing *hsp3102*, *hsp3103*, *hsp3104* or *hsp3105* tagged with a C-terminal c-Myc epitope tag at their genomic loci for Western blot analysis, the 5′ upstream and 3′ downstream of these genes were PCR-amplified, and cloned into the *Sal*I-*Sma*I and the *Sac*I-*Sac*II sites of pFA6a-13myc-kanMX6 [26], respectively. PCR primers are available upon request. All constructs were verified by PCR.

**Table 1:** List of *S*. *pombe* strains used in this study.

Strain	Genotype	Source
yHL6381	*h* ^*+*^ *his3-D1 leu1-32 ura4-D18 ade6-M210*	H. Levin
ySY1	*h* ^*+*^ *his3-D1 leu1-32 ura4-D18 ade6-M210 GFP-atg8*::*leu1*	This study
ySY2	*h* ^*+*^ *his3-D1 leu1-32 ura4-D18 ade6-M210* Δ*hsp3101*::*his3 GFP-atg8*::*leu1*	This study
ySY3	*h* ^*+*^ *his3-D1 leu1-32 ura4-D18 ade6-M210* Δ*hsp3102*::*kanMX6 GFP-atg8*::*leu1*	This study
ySY4	*h* ^*+*^ *his3-D1 leu1-32 ura4-D18 ade6-M210* Δ*hsp3103*::*kanMX6 GFP-atg8*::*leu1*	This study
ySY5	*h* ^*+*^ *his3-D1 leu1-32 ura4-D18 ade6-M210* Δ*hsp3104*::*kanMX6 GFP-atg8*::*leu1*	This study
ySY6	*h* ^*+*^ *his3-D1 leu1-32 ura4-D18 ade6-M210* Δ*hsp3105*::*kanMX6 GFP-atg8*::*leu1*	This study
ySY7	*h* ^*+*^ *his3-D1 leu1-32 ura4-D18 ade6-M210* Δ*sdj1*::*kanMX6 GFP-atg8*::*leu1*	This study
DY3510	*h* ^*+*^ *leu1-32 CFP-atg8*::*leu1*	L. L. Du
Zd299	*h* ^*+*^ *leu1-32 his3-D1* Δ*atg5*::*kanMX6*	L. L. Du
ySY8	*h* ^*+*^ *leu1-32* Δ*hsp3101*::*kanMX6 CFP-atg8*::*leu1*	This study
ySY9	*h* ^*+*^ *leu1-32* Δ*hsp3102*::*kanMX6 CFP-atg8*::*leu1*	This study
ySY10	*h* ^*+*^ *leu1-32* Δ*hsp3103*::*kanMX6 CFP-atg8*::*leu1*	This study
ySY11	*h* ^*+*^ *leu1-32* Δ*hsp3104*::*kanMX6 CFP-atg8*::*leu1*	This study
ySY12	*h* ^*+*^ *leu1-32* Δ*hsp3105*::*kanMX6 CFP-atg8*::*leu1*	This study
ySY13	*h* ^*+*^ *leu1-32* Δ*sdj1*::*kanMX6 CFP-atg8*::*leu1*	This study
ySY14	*h* ^*+*^ *leu1-32* Δ*atg5*::*kanMX6 CFP-atg8*::*leu1*	This study
ySY15	*h* ^*+*^ *his3-D1 leu1-32 ura4-D18 ade6-M210* Δ*atg5*::*kanMX6 GFP-atg8*::*leu1*	This study
yTW1	*h* ^*+*^ *his3-D1 leu1-32 ura4-D18 ade6-M210 hsp3102-myc*::*kanMX6*	This study
yTW2	*h* ^*+*^ *his3-D1 leu1-32 ura4-D18 ade6-M210 hsp3103-myc*::*kanMX6*	This study
yTW3	*h* ^*+*^ *his3-D1 leu1-32 ura4-D18 ade6-M210 hsp3104-myc*::*kanMX6*	This study
yTW4	*h* ^*+*^ *his3-D1 leu1-32 ura4-D18 ade6-M210 hsp3105-myc*::*kanMX6*	This study
ySY16	*h* ^*+*^ *his3-D1 leu1-32 ura4-D18 ade6-M210* Δ*atf1*::*ura4*	This study
ySY17	*h* ^*+*^ *his3-D1 leu1-32 ura4-D18 ade6-M210* Δ*sty1*::*ura4*	This study
ySY18	*h* ^*+*^ *his3-D1 leu1-32 ura4-D18 ade6-M210* Δ*atf1*::*ura4 hsp3102-myc*::*kanMX6*	This study
ySY19	*h* ^*+*^ *his3-D1 leu1-32 ura4-D18 ade6-M210* Δ*atf1*::*ura4 hsp3105-myc*::*kanMX6*	This study
ySY20	*h* ^*+*^ *his3-D1 leu1-32 ura4-D18 ade6-M210* Δ*sty1*::*ura4 hsp3102-myc*::*kanMX6*	This study
ySY21	*h* ^*+*^ *his3-D1 leu1-32 ura4-D18 ade6-M210* Δ*sty1*::*ura4 hsp3105-myc*::*kanMX6*	This study

*S*. *pombe* strains were grown in rich yeast extract supplements medium (YES) or synthetic Edinburgh minimal medium (EMM) [27]. Standard protocols for genetic manipulation of fission yeast were used [28].

### Plasmid construction

For overexpression of genes in *S*. *pombe*, *hsp3101*-*hsp3105* and *Sdj1* were PCR-amplified from the genomic DNA, cloned into the *Bam*H I and *Sma* I sites of the *S*. *pombe* expression plasmid pREP3X [29].

### Quantitative real-time RT-PCR

RNA was isolated from wild type *S*. *pombe* cells (yHL6381) using an E.Z.N.A.^®^ Yeast RNA Kit (OMEGA). Contaminating genomic DNA in RNA was removed by treatment with RNase-free DNase (Fermentas). RNA samples were reversed transcribed with the oligo (dT)_16_ primer using RevertAid^TM^ First Strand cDNA Sythesis Kit (Fermentas). Comparative qPCR analysis was performed using the StepOne^TM^ RT-PCR system (Applied Biosystems) with each primer sets (S1 Table). All reactions were performed in triplicate. Data analysis was performed by StepOne^TM^ software. The C_T_ values were normalized against actin mRNA levels from the same preparations to give the ΔC_T_ values. Fold changes in gene expression at different growth phases compared to the early exponential phase (OD_600_ = 0.5) were calculated by using the 2^-∆∆CT^ method.

### CFP-Atg8 processing assay

Cell lysates were prepared by alkaline extraction [30]. Total proteins were separated by 12% SDS/PAGE and immunoblotted with anti-GFP antibody (Roche).

### Western blot analysis

*S*. *pombe* whole cell extracts were prepared by bead beating using a FastPrep-24 machine (MP Biomedicals) [30, 31]. Proteins were separated by 15% SDS/PAGE and analyzed by immunoblotting using anti-Hsp3101 antibody (1:5000), anti-c-Myc antibody (1:5000) to detect Hsp3102-Myc, Hsp3103-Myc and Hsp3104-Myc, anti-Sdj1 antibody (1:5000) and anti-Sla1 antibody (1:5000) as a loading control. IRDye 800CW conjugated goat anti-rabbit IgG (LI-COR Biosciences) was used as secondary antibody. The fluorescent bands were detected using an Odyssey near-infrared fluorescence scanner (LI-COR Biosciences).

### Fluorescence microscopy

To detect the localization of GFP-Atg8 in wild type and mutant strains at different stages of growth, yeast strains expressing GFP-Atg8 from the promoter of Atg8 were grown in YES supplemented with 225 mg/l leucine, histidine, uracil and adenine at 30°C. Cells were harvested at different time points and cell pellets were suspended in PBS. The green fluorescence of EGFP was detected using λ_ex_ of 488 nm. All images were obtained on a Zeiss Axio Imager A1 microscope equipped with a PCO Sensicam CCD (charge-coupled-device) camera, and data were analyzed using MetaMorph image processing software (Universal Imaging).

### Survival Assays

Cell viability was measured by counting colonies generated by viable individual cells. Cells were grown in YEPD media (0.5% yeast extract, 0.5% peptone and 1% glucose) overnight and diluted to an OD_600_ of 0.2 in fresh medium and incubated at 30°C. An aliquot of each culture at the indicated time points were taken, serially diluted and plated on YES plates. Sensitivity to oxidative stress was evaluated by spotting assays. Fresh colonies were grown in liquid YES or leucine-selective media overnight, serially diluted, normalized to an OD_600_ of 3.0, and 3 μl of cells were spotted on rich YES or leucine-free minimal EMM plates without or with concentration of hydrogen peroxide. The plates were photographed after 5 days of incubation at 30°C.

## Results

### Deletion of *hsp3104* and *sdj1*, and to a lesser extent, *hsp3102* and *hsp3105*, leads to a delay in CFP-Atg8 processing in stationary phase

We investigated whether *S*. *pombe* homologs of DJ-1 have a role in autophagy. To this end, we employed the CFP-Atg8 processing assay which is widely used to monitor autophagy over time from yeast to human [32–34]. This assay is based on the fact that upon induction of autophagy, CFP-Atg8 is attached on the inner membrane of the autophagosome, delivered to the vacuole and processed, generating the protease-resistant free CFP. Thus, the protein level of CFP detected by Western blot can be used to estimate the autophagic activity. We carried out systematic gene disruptions of individual genes encoding DJ-1 homologs in a *S*. *pombe* strain expressing CFP-Atg8. All six genes could be disrupted individually without eliminating the growth of *S*. *pombe* cells.

**Fig 1:**
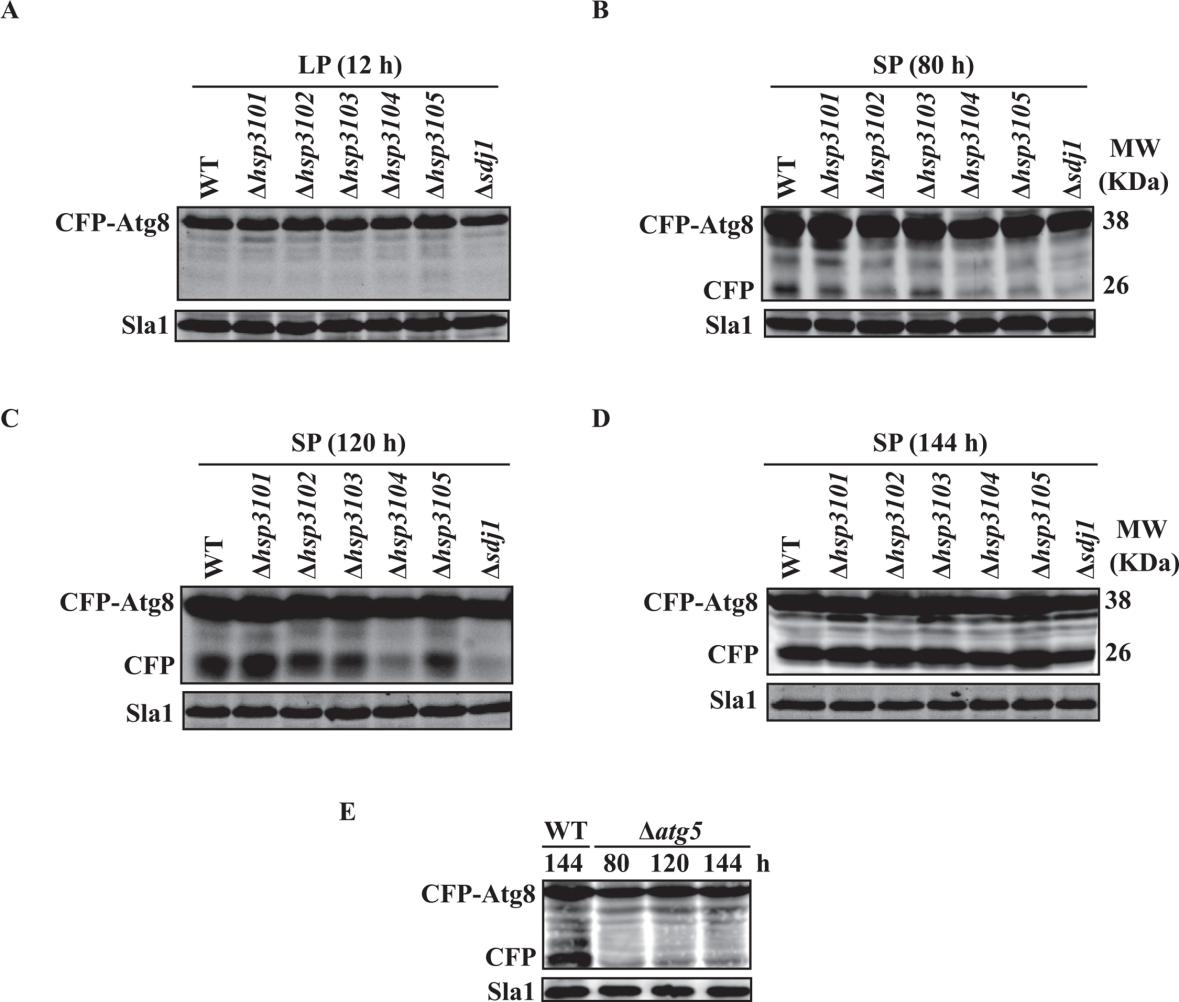
Loss of *hsp3102*, *hsp3104*, *hsp3105* and *sdj1* leads to delay of autophagy during stationary phase. *S*. *pombe* cells expressing CFP-Atg8 were grown in YES medium and harvested at logarithmic phase (LP) (A) and prolonged stationary phase (SP) (B-D). (E) Deletion of *atg5* abolished CFP-Atg8 processing. Cells were lysed by sodium hydroxide and cell-free lysates were analyzed by immunoblotting using antibodies against GFP and Sla1 (loading control). The presence of free CFP is an indicator of autophagic flux.

We first examined autophagy in single-gene deletion mutants of all six *S*. *pombe DJ-1*-relataed genes during growth under rich-medium growth conditions. In the logarithmic growth phase (Log phase), only one protein band corresponding to CFP-Atg8 was detected in the wild-type and all deletion mutants (Fig 1A). Free CFP was detected in wild-type and all deletion mutants during prolonged stationary phase (Fig 1). However, the appearance of free CFP was considerably delayed in Δ*hsp3104* and Δ*sdj1* mutants and, to a lesser extent, in Δ*hsp3102* and Δ*hsp3105* mutants compared to the wild-type strain (Fig 1B and 1C). At 144 h time point, the level of free CFP in all deletion mutants was comparable to that of the wild-type strain. Deletion of autophagy-related gene *atg5*, which is necessary for autophagy, in the wild-type and deletion mutant background completely abolished CFP-Atg8 processing (Fig 1E), indicating that CFP-Atg8 processing in stationary-phase cells is the ATG-dependent autophagic process. Altogether, these results indicated that Hsp3104 and Sdj1 and, a lesser extent, Hsp3102, Hsp3105 are involved in, but not essential for the normal autophagy in prolonged stationary phase.

Since fission yeast autophagy is induced by nitrogen starvation but not carbon starvation and rapamycin treatment [35], we next examined whether *S*. *pombe* DJ-1 homologs play a role in autophagy under nitrogen starvation conditions. Autophagy occurred at a basal level under normal growth conditions (data not shown). Under nitrogen starvation conditions, both wild-type strain and the deletion mutants showed a significant increased level of CFP-Atg8. In addition, the *DJ-1*-related gene deletion mutants had levels of autophagy similar to that of the wild-type strain (data not shown).

### *S*. *pombe* DJ-1 homologs are involved in the localization of Atg8 at PAS

**Fig 2:**
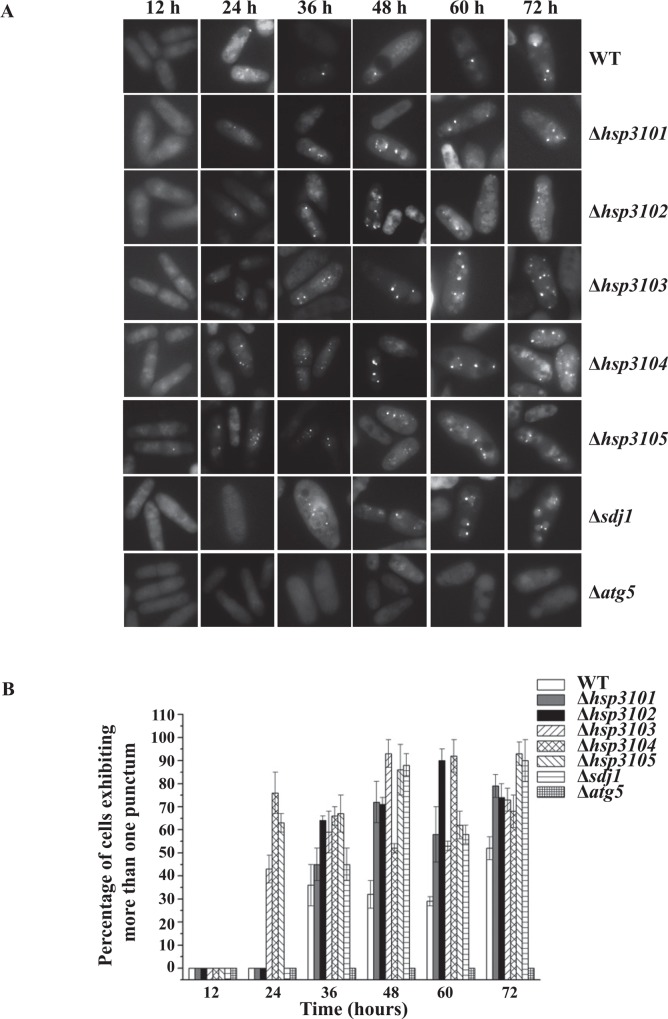
Deletion of *hsp31* and *sdj1* genes affects subcellular localization of GFP-Atg8. (A) GFP-Atg8 localization in wild type and deletion mutants of *hsp31* and *sdj1*. Cells were grown in YES medium and analyzed by fluorescence microscopy. Samples were taken at indicated time points during growth. (B) Quantitative analysis of the GFP-Atg8 puncta in the panel (A) (mean ± SEM; n>500 cells). Statistical significance (*P < 0.05) was determined by Student's *t* test.

Next, we examined whether *S*. *pombe* DJ-1 homologs play a role in the localization of Atg8 at the phagophore assembly site/pre-autophagosomal structure (PAS), which is a unique site where most autophagy-related proteins act together to form the double-membraned vesicles known as autophagosomes. Since GFP gives a brighter signal than CFP in our hands, we integrated a GFP tag at the N-terminus of endogenous Atg8 in a wild-type strain and all deletion mutants of *S*. *pombe* DJ-1 homologs. Fluorescence microscopy revealed that GFP-Atg8 was dispersed in the cytoplasm during log phase. After entering stationary phase, a few GFP-Atg8 punctate signals corresponding to the PAS were readily detected in wild type cells and all deletion mutants but not in the *atg5* deletion mutant (Fig 2A). However, the detection of the GFP-Atg8 puncta in Δ*sdj1* cells were 12 h later than wild-type and other deletion mutants (Fig 2A). In addition, all deletion mutants of *S*. *pombe* DJ-1 homologs accumulated more GFP-Atg8 puncta than the wild type cells (Fig 2A). Similar results were observed in deletion mutants of *S*. *pombe* autophagy-related genes *atg2*, *atg18b* and *atg18c* [23]. Quantitative analysis of GFP-Atg8 puncta in the wild-type and deletion mutants of *S*. *pombe* DJ-1 homologs is summarized in Fig 2B.

### *sdj1* deletion resulted in increased sensitivity to H_2_O_2_, while *sdj1* overexpression led to increased resistance to H_2_O_2_.

**Fig 3:**
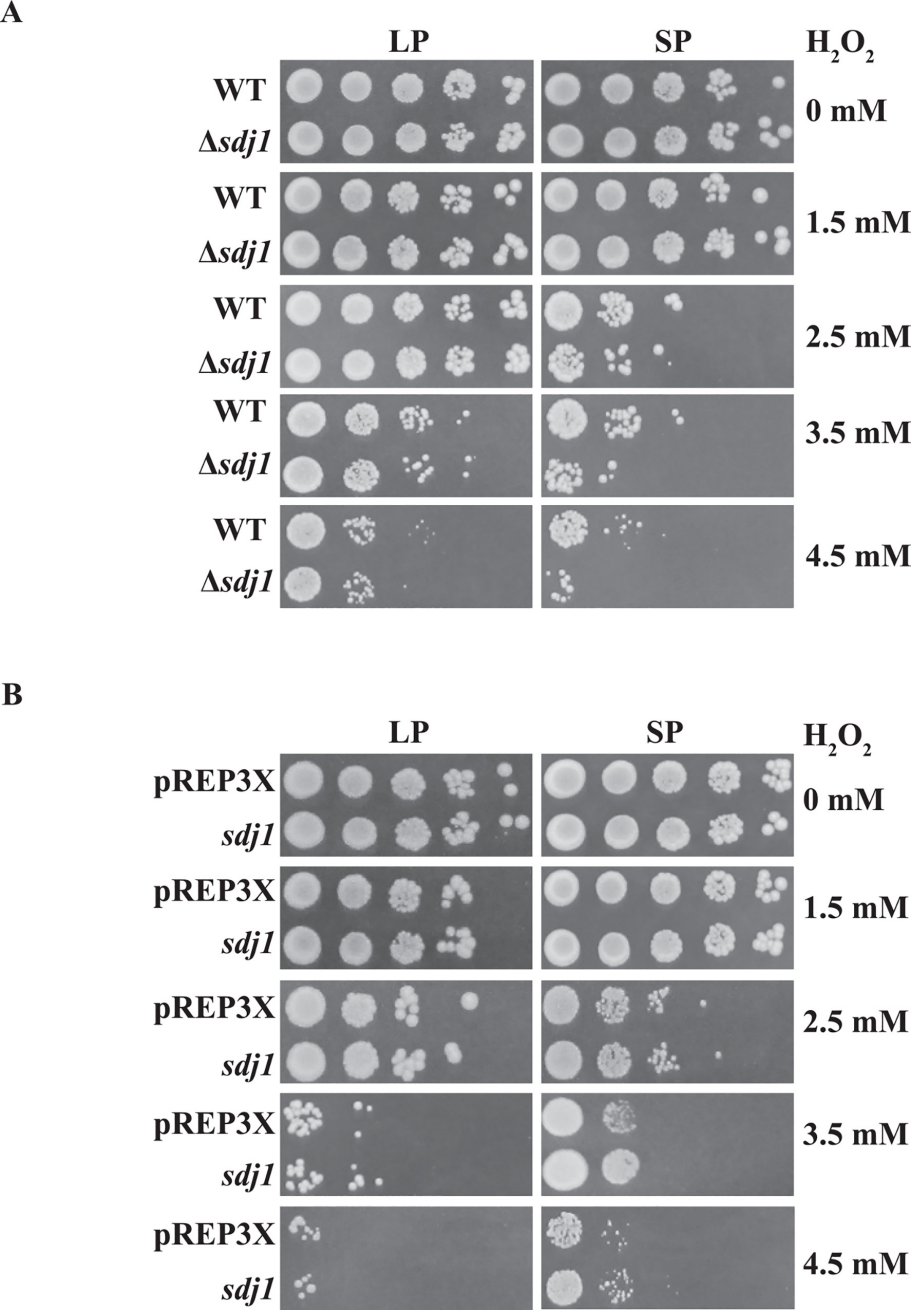
Sdj1 is involved in antioxidant defense in *S*. *pombe*. (A) Deletion of *sdj1* increases the H_2_O_2_ sensitivity of cells. (B) Overexpression of *sdj1* decreases the sensitivity of stationary-phase wild-type cells to H_2_O_2_. Wild-type and Δ*sdj1* cells were grown to logarithmic phase (LP) or stationary phase (SP) in YES medium. An aliquot of each culture was serially 10-fold diluted and spotted onto YES or selective leucine-free minimal EMM plates without or with the indicated concentration of H_2_O_2_. Plates were incubated at 30°C.

Since DJ-1 is involved in oxidative stress, we were next interested in determining whether *S*. *pombe* Hsp3101-Hsp3105 and Sdj1 are also involved in resistance to hydrogen peroxide-induced oxidative stress. To this end, wild-type cells and deletion mutants of *S*. *pombe* DJ-1 homologs were grown to either log or stationary phase, and sensitivity to oxidant H_2_O_2_ were determined by spotting assays using H_2_O_2_ concentrations in a range of 0–4.5 mM. As shown in Fig 3, *sdj1* deletion did not alter log phase cell sensitivity to H_2_O_2_. However, the *sdj1* deletion mutant in stationary phase showed increased sensitivity to H_2_O_2_ compared to the wild-type strain. In contrast, other deletion mutants showed similar sensitivity to H_2_O_2_ compared to the isogenic wild-type strain (data not shown).

We next determined whether overexpression of *sdj1* in the wild-type cells could modulate the sensitivity of *S*. *pombe* cells to H_2_O_2_. The wild-type cells that overexpressed *sdj1* from the pREP3X *nmt1* promoter survived better than cells transformed with the empty pREP3X vector (Fig 3B). These results showed that overexpression of *sdj1* increased the survival of wild-type stationary-phase cells in the presence of exogenous H_2_O_2_.

### Deletion of *S*. *pombe* DJ-1 homologs reduces the survival of stationary phase cells

**Fig 4:**
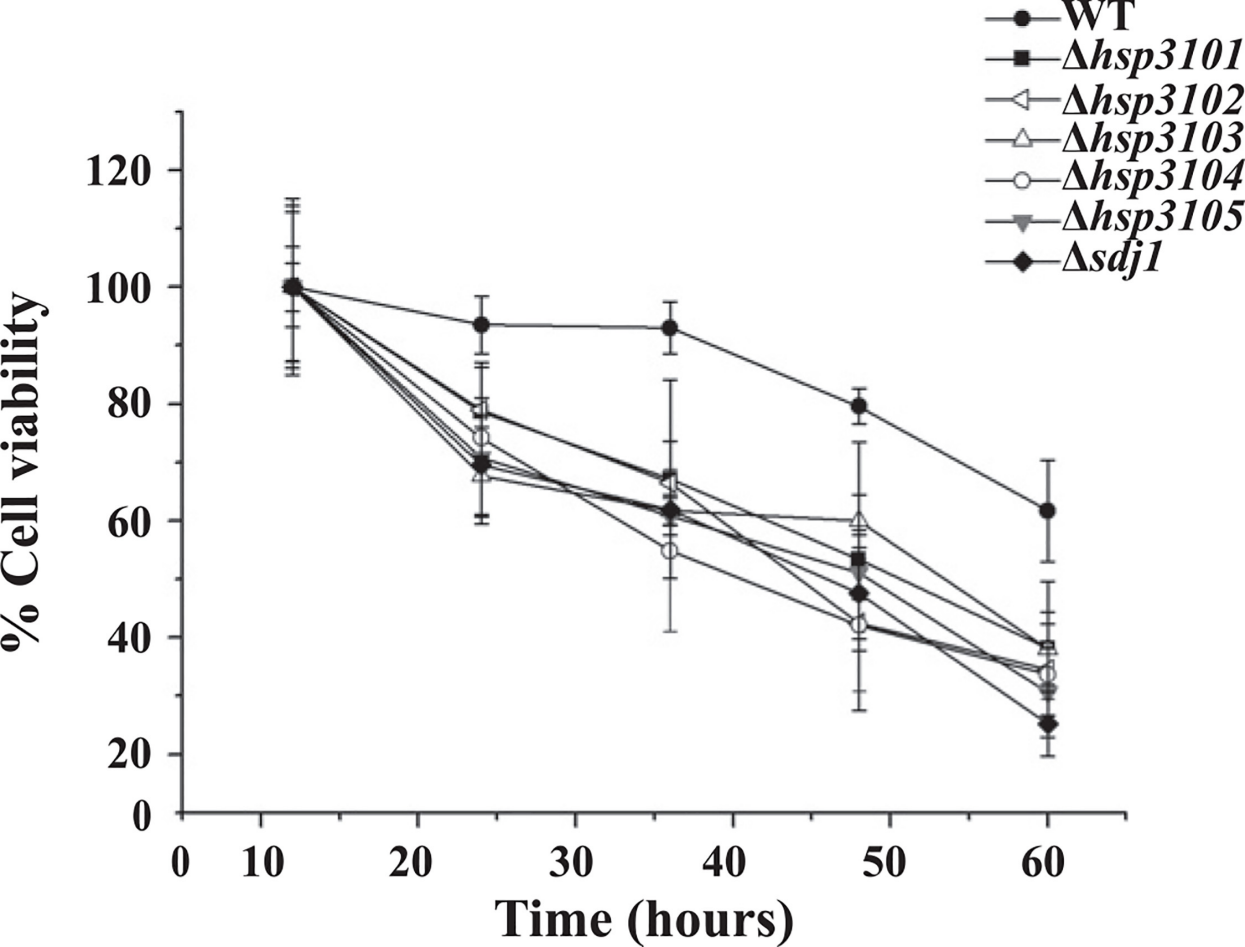
Deletion of *S*. *pombe* DJ-1 homologs reduces cell survival in stationary phase. Cells were grown in low-glucose rich YEPD medium (1% glucose). The survival difference between the wild-type and mutants is more noticeable when cells were grown in YEPD. At the indicated time points, an aliquot of each culture was taken, serially diluted and plated to obtain viable colonies. Viability of stationary phase cells was measured as the ability of cells to form colonies on rich medium plates. The graph shows the percentage of viable cells ± SEM. The viability of cells in exponential growth (12 h) was set to 100%.

In our prior study, we have previously shown that deletion of *hsp3101-hsp3104* and *sdj1* has no effect on cell growth under normal conditions [19]. Because the *S*. *pombe* homologs *of* DJ-1 appeared to function in stationary phase, we determined whether deletion of *S*. *pombe* DJ-1 homologs affected cell survival in stationary phase. When cells were grown in rich YES medium, there was no difference in survival between the wild-type and deletion mutants of *S*. *pombe* DJ-1 homologs (data not shown). Deletion of *S*. *pombe* homologs of DJ-1 resulted in reduced viability in stationary phase when cells were grown in low-glucose rich YEPD medium (1% glucose) (Fig 4).

### Genes encoding *S*. *pombe* DJ-1 homologs are up-regulated during stationary phase

**Fig 5:**
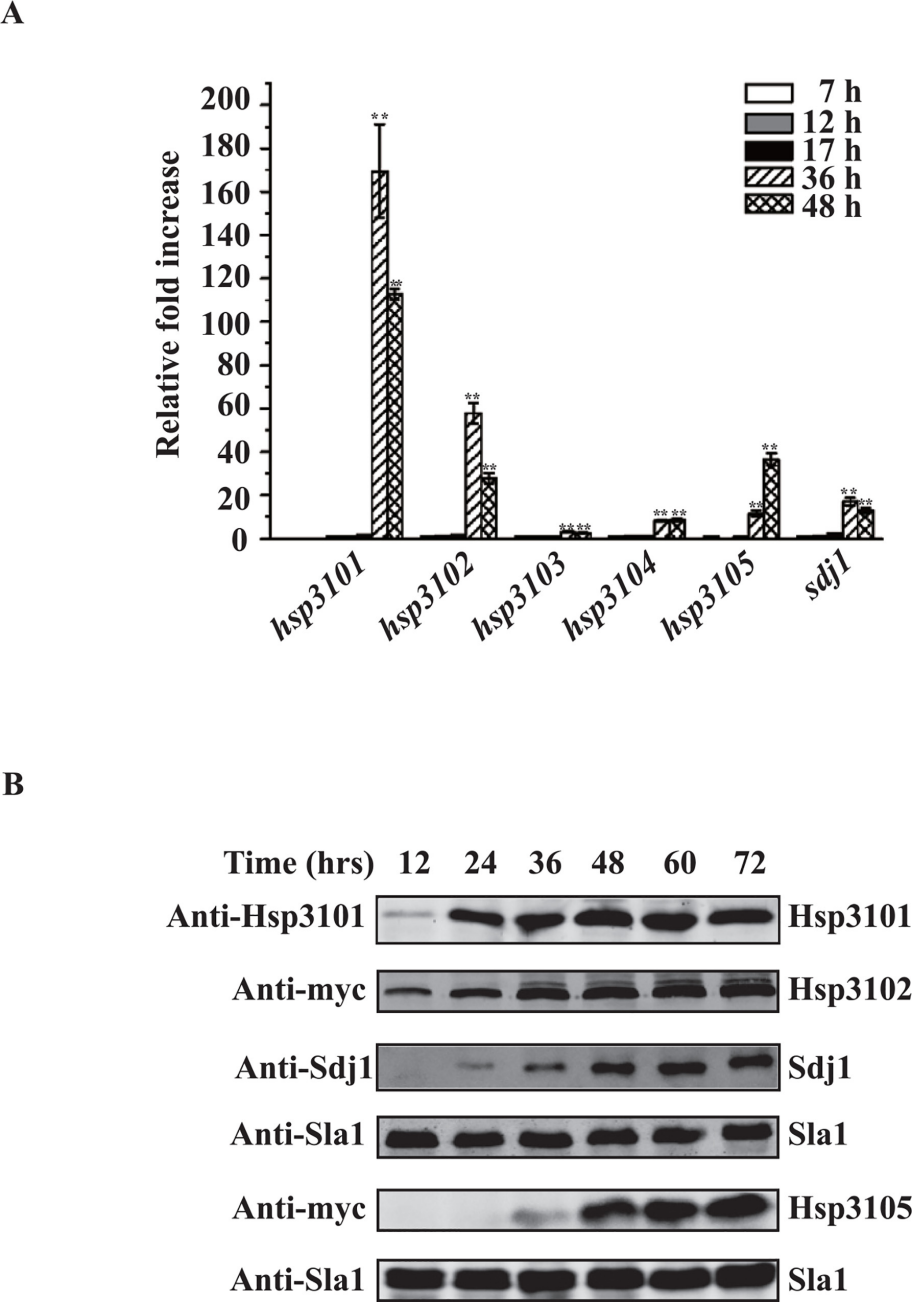
Induction of *S*. *pombe* homologs of DJ-1. (A) qRT-PCR analysis of expression of *S*. *pombe hsp3101-hsp3105* and *sdj1* genes in the wild-type cells. Total RNA was isolated from the wild-type cells grown in YES medium at the indicated time points. All mRNA levels were normalized to the control *act1* ^+^ mRNA level and were expressed as fold change relative to the mRNA levels at the 7 h time point, which was set at a value of 1. Data are presented as mean ± SD (*p* ≤0.01; *t* test). (B) Immunoblot analyses of Hsp3101, Hsp3102, Hsp3105 and Sdj1 expression in wild-type cells. Crude extracts were prepared from the wild-type cells at indicated time points (h). Total proteins were separated on SDS/PAGE gels and immunoblotted using anti-Hsp3101 Ab, anti- Sdj1 Ab, anti-Myc Ab, which detects Hsp3102-Myc and Hsp3105-Myc, and anti-Sla1 Ab (serves as a loading control).

Because *S*. *pombe* homologs *of DJ-1* seem to function in stationary phase, we asked whether their expression was induced in stationary phase. To this end, we first determined the relative mRNA levels of the genes encoding *S*. *pombe* DJ-1 homologs using quantitative RT-PCR (qRT-PCR) and primers specific for each *S*. *pombe* DJ-1 homolog. As shown in Fig 5A, expression of all *S*. *pombe* DJ-1 homologs was up-regulated at stationary phase. *hsp3101* and *hsp3102* were induced ~170-fold and ~58-fold, respectively, at the 36 h time point and were the most highly induced genes in the stationary phase. In contrast, *hsp3103* and *hsp3104* were induced only ~3-fold and ~9-fold, respectively (Fig 5A). *hsp3105* and *sdj1* were induced ~37-fold and ~17-fold, respectively.

To validate the results of qRT-PCR, we examined whether the protein levels for *S*. *pombe* Hsp3101-Hsp3105 and Sdj1 were increased in stationary phase. Because only antibodies against Hsp3101 and Sdj1 are available, we generated four strains that endogenously expressed C-terminally thirteen-Myc-tagged Hsp3102-Hsp3105 in the wild type strain yHL6381. In agreement with the qRT-PCR data, Western blot analysis revealed that the proteins levels for Hsp3101, Hsp3102, Hsp3105 and Sdj1 were significantly up-regulated in stationary phase (Fig 5B). However, despite our efforts, we were unable to detect Hsp3103-Myc and Hsp3104-Myc by Western blot using anti-Myc antibody, most likely due to very low levels of expression of these two proteins. These results suggest that the *S*. *pombe* homologs of DJ-1 are functionally associated with the stationary phase.

**Fig 6:**
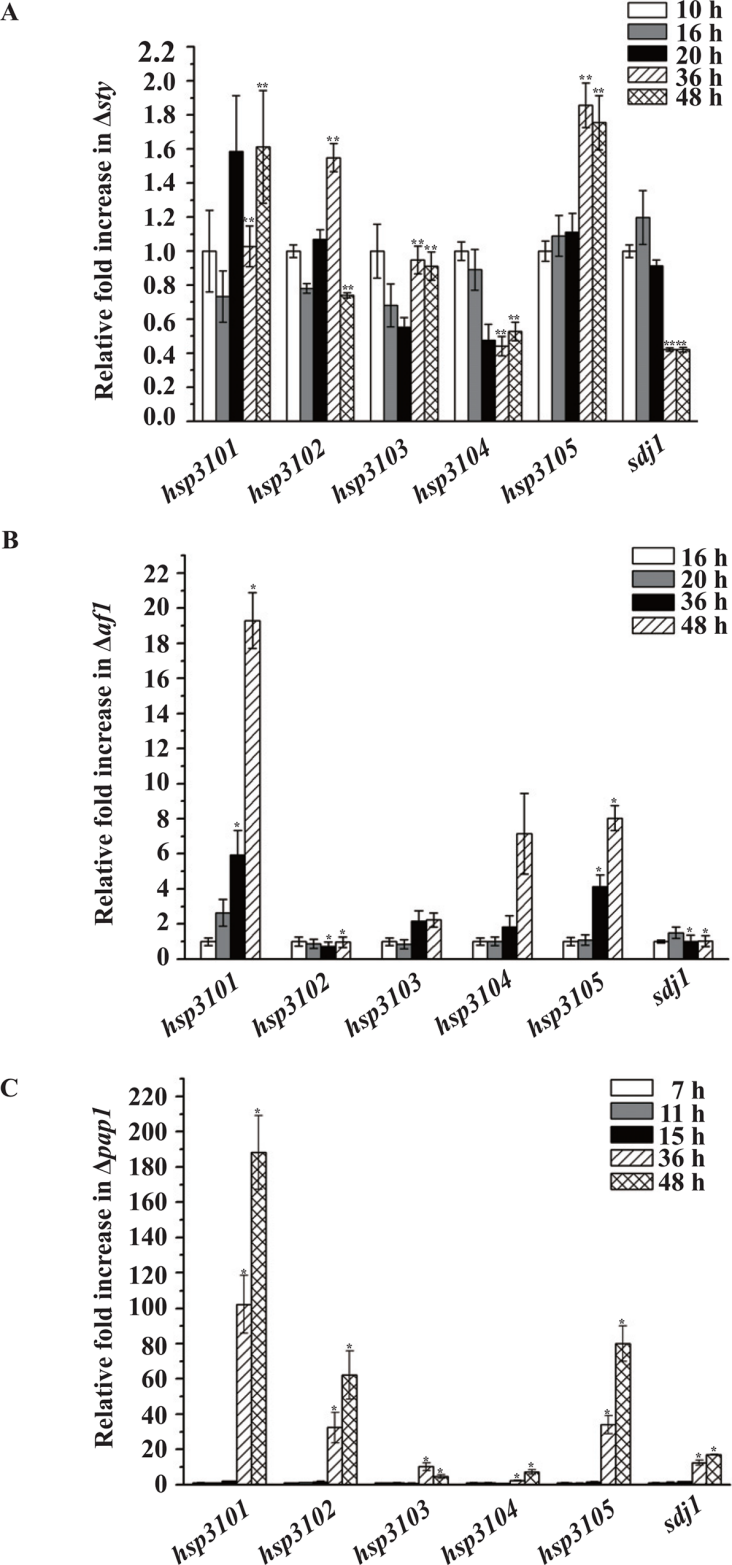
Expression levels of *S*. *pombe DJ-1* homologs in wild-type, Δ*sty1*, Δ*atf1* and Δ*pap1* cells. *S*. *pombe hsp3101*-*hsp3105* and *sdj1* in Δ*sty1* (A), Δ*atf1* (B) and Δ*pap1* (C) were grown in YES medium, and total RNAs were extracted at indicated times and analyzed by qRT-PCR. Columns indicate mean values of at least three independent experiments; error bars represent standard error of the mean (SEM) (n = 3, separate experiments). Statistical analyses were performed using Student *t* test (*p < 0.05, **p < 0.01). Reliable data could not be obtained beyond the 48 h time point due to the loss of viability caused by *sty1 or atf1* depletion.

We next tested whether the stress-activated MAP kinase Sty1 (also known as Spc1), which plays an essential role in the cellular response to nutrient deprivation and environmental stresses, was required for the induction of *S*. *pombe* DJ-1 homologs. To this end, we disrupted *sty1* in the wild-type yHL6381 and measured the mRNA expression levels of *S*. *pombe hsp31* genes and *sdj1* in Δ*sty1*. Consistent with the previous findings that deletion of any component of the Spc1-Atf1 pathway causes rapid loss of cell viability after cells enter stationary phase [36], Δ*sty1* cells lost viability dramatically in stationary phase (data not shown). It was very difficult to isolate high quality RNA after 48 h incubation due to RNA degradation. Deleting *sty1* almost completely abolished the stationary phase induction of *S*. *pombe hsp3101*-*hsp3105* and *sdj1* (Fig 6A). Since Sty1 regulates stress-dependent transcription, at least in part, through the basic-region leucine-zipper (bZIP) transcription factor Atf1 [37, 38], we constructed an Δ*atf1* strain and found that induction of *hsp3102* and *sdj1* expression was essentially abolished in Δ*atf1* cells, whereas induction of *hsp3101* and *hsp3105* was attenuated (Fig 6B). *hsp3101* and *hsp3105* expression was induced ~19-fold and ~8-fold, respectively, at 48 h time point (Fig 6B). By contrast, induction of *hsp3103* and *hsp3104* expression appeared to be unaffected in Δ*atf1* cells (Fig 6B).

Besides the Sty1 pathway, the Pap1-mediated regulatory pathway is required for the expression of genes involved in the adaptation to oxidative stress in fission yeast [39]. To determine if Pap1 is involved in the induction of *S*. *pombe DJ-1* homologs, we deleted *pap1* in the wild-type strain. Deletion of *pap1* did not affect the induction of *S*. *pombe DJ-1* homologs (Fig 6C).

**Fig 7:**
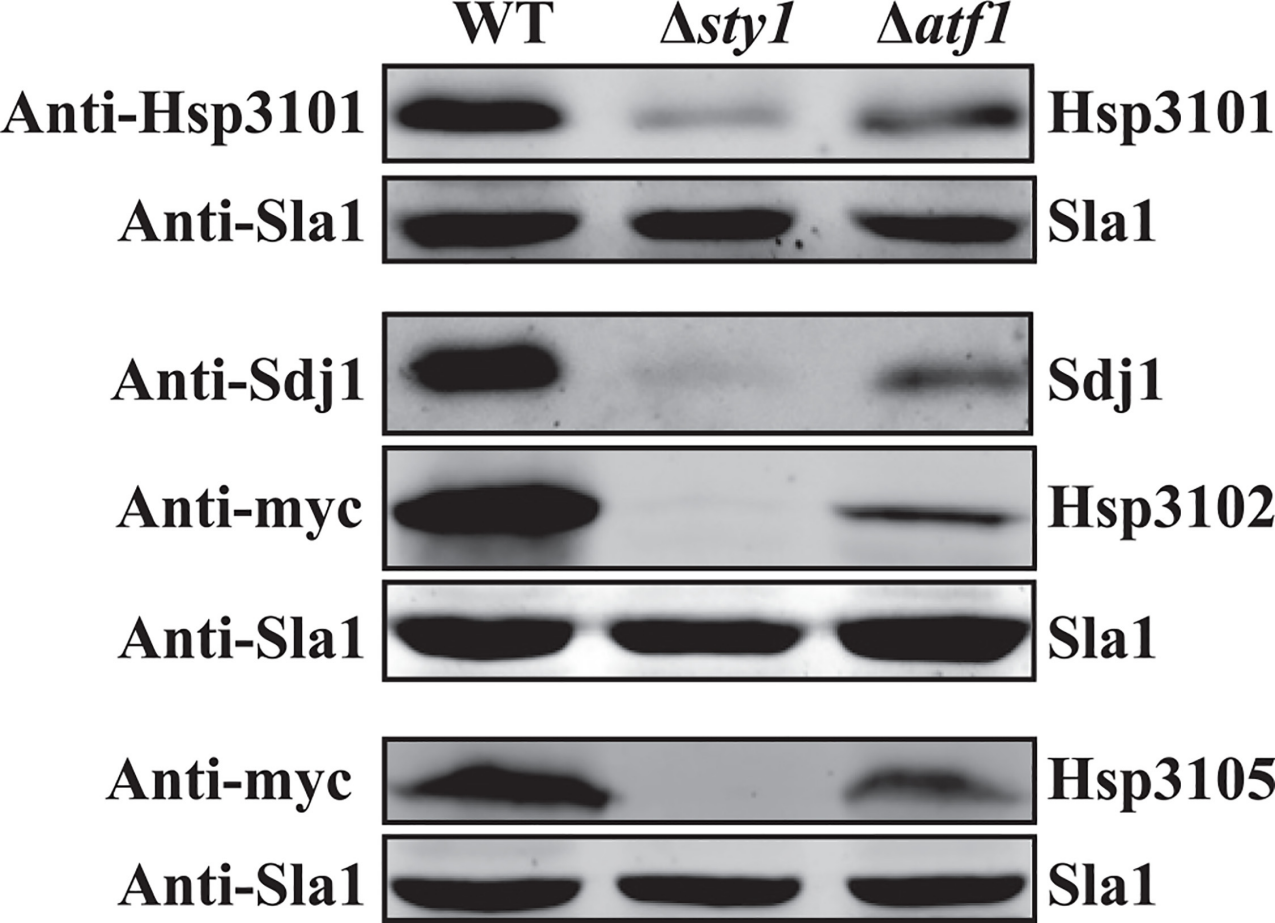
Western blot analysis of Hsp3101, Hsp3102, Hsp3105 and Sdj1 in wild-type, Δ*sty1* and Δ*atf1* cells. Wild-type, Δ*sty1* and Δ*atf1* cells were grown in YES medium. Cell lysates were prepared, and proteins were separated by SDS/PAGE and analyzed by immunoblotting with indicated antibodies. Sla1 serves as the loading control.

To verify these results, we performed Western blotting with antibodies against Hsp3101 and Sdj1, and anti-Myc antibody to detect Hsp3102 and Hsp3105. Consistent with the qRT-PCR results, deletion of either *sty1* or *atf1* resulted in the reduction of Hsp3101, Hsp3102, Hsp3105 and Sdj1 protein levels (Fig 7).

We analyzed the 5′ upstream regions of *S*. *pombe hsp31* genes and *sdj1* to understand how these genes are activated. We found that the promoter regions of *hsp3101*-*hsp3103* and *sdj1* possess a core environmental stress response (CESR) motif, which is homologous to the ATF/CRE motif and is found in *S*. *pombe* genes in response to stress condition [40] (S2 Table). In addition, the promoter region of *sdj1* contains an ATF/CRE motif, which is the binding site for the ATF/CREB family of transcription factors, and is required for stress-dependent activation of transcription [36, 38]. These motifs are candidate Aft1 binding sites [40].

## Discussion

Stationary phase cells undergo major metabolic and transcriptional reprogramming to adapt to nutrient starvation and various stress conditions due to accumulation of toxic metabolites, characteristic of stationary-phase cultures. We have demonstrated that in *S*. *pombe* under normal growth conditions, autophagy is induced in prolonged stationary phase. Induced autophagy may provide a way for cells in prolonged stationary phase to overcome nutrient-depletion conditions. Deletion of *hsp3102*-*hsp3105* and *sdj1* delays vacuolar degradation of CFP-Atg8, as indicated by delayed proteolytic cleavage of CFP-Atg8. Further, the localization of Atg8 at PAS appears to be influenced by *S*. *pombe* Hsp3101-Hsp3105 and Sdj1 proteins. The number of GFP-Atg8 puncta is increased in all deletion mutants of *S*. *pombe* DJ-1 homologs. Generation of Atg8 puncta is somewhat delayed in the Δ*sdj1* deletion mutant. Thus, in *S*. *pombe*, individual DJ-1 homologs appear to be involved in, but are not absolutely required for the execution of autophagy in prolonged stationary phase. However, we cannot rule out the possibility that the deletion of all *S*. *pombe* homologs of DJ-1 may have a synergistic effect on autophagy. Addressing this possibility would require the generation of a mutant strain in which all *S*. *pombe* DJ-1 homologs are deleted. However, we failed to obtain such a mutant strain due to technical difficulties. In addition, *S*. *pombe* DJ-1 homologs are not involved in autophagy under nitrogen starvation conditions (data not shown). These results are in sharp contrast to the situation in *S*. *cerevisiae*, where deletion of each of *HSP31* genes severely impairs autophagy during stationary phase and under carbon starvation conditions, indicating that each *S*. *cerevisiae HSP31* gene is required for autophagy under these conditions.

Autophagy protein Atg13 plays a central role in the initial step of PAS formation and Atg13 phosphorylation is crucial for autophagy in *S*. *cerevisiae* cells [41]. As Atg13 phosphorylation has been shown to be altered in *S*. *cerevisiae* Δ*HSP31* cells [16], we tried to examine whether there was an alteration in phosphorylated Atg13 in deletion mutants of *S*. *pombe* DJ-1 homologs. However, despite our efforts, we could not detect any endogenous Atg13 protein in *S*. *pombe* cells grown in the late stationary phase by Western blotting likely due to an extremely low level of endogenous Atg13 expression in *S*. *pombe* (data not shown). This is consistent with a previous report, which showed that endogenous Atg13 was barely detected in *S*. *pombe* cells grown under nitrogen starvation conditions [35].

In fission yeast, the Sty1 stress-activated protein kinase pathway, which is similar to the *S*. *cerevisiae* Hog1 pathway and the mammalian p38 MAP kinase pathway, plays a critical role in regulating transcriptional responses to nutrient starvation and other stresses [42, 43]. In this study we show that all *S*. *pombe* DJ-1 homologs are induced in stationary phase, albeit *hsp3103* and *hsp3104* are induced to a much lesser extent. Furthermore, the induction of these genes are dependent on the mitogen-activated protein kinase (MAPK) Sty1. Interestingly, deletion of *atf1*, which is the main substrate of Sty1 and regulates a large set of stress response genes, essentially abolishes *hsp3102* and *sdj1* induction, but does not affect the induction of *hsp3103* and *hsp3104* and only partially abolishes induction of *hsp3101* and *hsp3105* expression. Thus, the induction of *hsp3102* and *sdj1* is solely dependent on Aft1; *hsp3103* and *hsp3104* are induced by transcription factor (s) other than Aft1; the full induction of *hsp3101* and *hsp3105* expression requires Atf1 and additional transcription factor (s). Consistent with our results, the Pap1-mediated regulatory pathway is not involved in the induction of *S*. *pombe* DJ-1 homologs.

It is important to note that expression of *S*. *pombe* DJ-1 homologs is also induced by other stresses. A global analysis of transcriptional response of fission yeast to environment stresses shows that the expression of *hsp3101*, *hsp3102*, *hsp3104* and *sdj1* are increased during cellular response to oxidative stress, osmotic stress, heat stress, and heavy metal stress (i.e., treatment with cadmium sulfate) [40]. In addition, a genome-wide analysis of gene expression under nitrogen-limiting reveals that *hsp3105* expression is increased during nitrogen depletion [44].

Our previous and current data suggest that *S*. *pombe* DJ-1 homologs are involved in the stationary-phase stress responses. This conclusion is based on the following observations. First, *S*. *pombe* Hsp3101 and Hsp3102 are the major GSH-independent glyoxalase III that may have some role in protecting cells from reactive carbonyl species toxicity accumulated during the stationary phase of yeast growth [19]. Second, *S*. *pombe* DJ-1 homologs appear to be involved but not essential for autophagy in stationary phase. Third, Sdj1 is likely to play a role in defense against oxidative stress during stationary phase. Fourth, expression of *S*. *pombe* DJ-1 homologs are induced in stationary phase in a Sty1-dependent manner. Interestingly, *S*. *pombe* DJ-1 homologs exhibit different patterns of subcellular localization [19]. Moreover, induction of *S*. *pombe* DJ-1 homologs may involve different transcription factors. Based on these observations, we speculate that *S*. *pombe* DJ-1 homologs may play overlapping yet distinct roles in stress responses.

## Supporting Information

S1 Table Nucleotide sequences of primers for qRT-PCR analysis. (DOCX)

S2 Table Identification of the CESR and ATF/CRE motifs in *S*. *pombe* homologs of *DJ-1*. The CESR motif: TKACGT, where K is T or G; the ATF/CRE motif: KWCGTCA, where K is T or G and W is T or A.
(DOCX)
