# Supplementary material for: Schizosaccharomyces pombe Homologs of Human DJ-1 Are Stationary Phase-Associated Proteins That Are Involved in Autophagy and Oxidative Stress Resistance
Source: PLoS One. 2015 Dec 1;10(12):e0143888. doi: 10.1371/journal.pone.0143888 (PMC4666628; doi:10.1371/journal.pone.0143888)
Supplement: S1 Table — (DOCX) [file pone.0143888.s001.docx]

**S1 Table. Nucleotide sequences of primers for qRT-PCR analysis.**

| Gene | Forward primer (5′ to 3′) | Reverse primer (5′ to 3′) |
| --- | --- | --- |
| *act1* | AAGGCTAGCTCTGCATTCGTCTAT | TCCGCTCTTAACATCTCATGAGG |
| *SpDJ-1* | GATTTCTAGAAGCCTCGGACTCG | TCTAGTATAACAGGTGCGAGACCG |
| *hsp3101* | GGTCATGCAGCCATGTTTGATC | AGGCAGCATAACTGGACCGTG |
| *hsp3102* | GGCGGTAGCAAGAGAAGTGTTTAC | TGCCTTCAACTGATTGTGGATTC |
| *hsp3103* | TGGCCGAGGATAAATCCAAAC | CACCGAATGGGTTTGGTGAC |
| *hsp3104* | GTAGGATTTGTGTTCATAATGCCTTG | GCGTTTAGAGTTTCATTTGTTATGGAT |
| *hsp3105* | TCTACCGCCTCTGTGAAAGAGAAT | GCAGGGCACATCAATGTCTGTC |
