# Supplementary material for: Schizosaccharomyces pombe Homologs of Human DJ-1 Are Stationary Phase-Associated Proteins That Are Involved in Autophagy and Oxidative Stress Resistance
Source: PLoS One. 2015 Dec 1;10(12):e0143888. doi: 10.1371/journal.pone.0143888 (PMC4666628; doi:10.1371/journal.pone.0143888)
Supplement: S2 Table — The CESR motif: TKACGT, where K is T or G; the ATF/CRE motif: KWCGTCA, where K is T or G and W is T or A. (DOCX) [file pone.0143888.s002.docx]

**S2 Table. Identification of the CESR and ATF/CRE motifs in *S. pombe* homologs of *DJ-1*.**

| Gene | CESR | | ATF/CRE | |
| --- | --- | --- | --- | --- |
|  | Sequence | bp from the motif to the ATG start codon | Sequence | bp from the motif to the ATG start codon |
| *hsp3101* | TTACGT | 108 |  |  |
| *hsp3102* | TGACGT | 446 |  |  |
| *hsp3103* | TTACGT | 169 |  |  |
| *hsp3104* |  |  |  |  |
| *hsp3105* |  |  |  |  |
| *SpDJ-1* | TTACGT | 1272 | TACGTCA | 117 |
